# Monthly Report of Diseases

**Published:** 1800-06

**Authors:** 


### MONTHLY REPORT of DISEASES,

Admitted under the Care of the PHYSICIANS of the FINSBURY DISPENSARY St. John's Square, Clerkenwell.

*The District, in which the [illegible] of the Finsbury Dispensary are visited, comprehends the Parishes of St. James, and of St. John, Clerkenwell; of St. Luke; of St. Sepulchre within and without; of St. Bartholomew, the Great and the Less; the Liberties of the Rolls, and of Glass-House Yard; the Town of Islington; the Parishes of St. Pancras; of St. Andrew, Holborn; and of St. George the Martyr, Queen's-square. This Tract of Ground may properly enough be termed, a North Western District of the Metropolis.*

LIST of DISEASES, &c. from April 20, to May 20.

| | No. of Cases. | | No. of Cases. |
|---|---|---|---|
| Continued Fever | 8 | Asthenia | 10 |
| Sore Throat | 2 | Cough and Dyspnœa | 16 |
| Pneumonia | 3 | Phthisis | 6 |
| Hæmoptysis | 2 | Paralysis | 4 |
| Dysentery | 3 | Hysteria | 5 |
| Diarrhœa | 7 | Jaundice | 2 |
| Chlorosis and Amenorrhœa | 16 | Dropsy | 9 |
| Menorrhagia | 12 | Schrophula | 6 |
| Leucorrhœa | 13 | Gout | 1 |
| Acute Rheumatism | 2 | Prurigo | 17 |
| Chronic Rheumatism | 14 | Cephalæa | 4 |
| Lumbago | 7 | Worms | 4 |
| Hypochondriasis and Dyspepsia | 12 | Infantile Diseases | 14 |
| | | Hæmorrhois | 2 |

The principal difference that is to be observed between the above list and that of the preceding month, is, that in consequence of a change of season, it exhibits a much smaller proportion of *pulmonary* diseases.

The weather, it may in general be remarked, has more influence upon complaints of the lungs than any remedies which are applied. That credit is accordingly too often given to the advice advice of the phyſician, which is, in fact, due to a favourable viciſſitude in the atmoſphere. This remark applies more eſpecially, to thoſe catarrhal affections which occur at an advanced period of life.

Perſons, at an advanced period of life, are peculiarly addicted to a ſuperſtitious reverence for medicines; and yet, it is to them that medicines are with the leaſt efficacy and propriety applied. The coughs and aſthmas of the aged are moſt frequently relieved by a change of air, even to a one leſs pure. It would be remarkable that change of air was not, in ſuch caſes, more generally preſcribed, if we did not reflect that air is not an article in an apothecary's ſhop. At the ſame time, although medicines are ſeldom uſeful to the aged, by acting immediately upon the body, they may however be, in ſome inſtances, eſſentially ſo, by acting upon the imagination.

Upon the influence of the imagination in curing diſeaſes, a judicious and ingenious pamphlet has been lately publiſhed by Dr. Haygarth, of Bath. It is a ſubject of great intereſt, and almoſt unbounded extent. The mind is continually meddling with the body, and interfering with the remedies which are applied to it. A due attention to this circumſtance would afford much inſtruction to phyſicians, and throw new light upon the efficiency of medical applications.

Even a kindneſs of manner on the part of a medical attendant, that beſpeaks an intereſt in his patient's health, may not unfrequently be conducive to its reſtoration; gratitude will cure a diſeaſe, when it is out of the reach of all other remedies. A patient will get well, or, which in many caſes amounts nearly to the ſame thing, will endeavour to fancy himſelf well, in order to *oblige* his phyſician. On the other hand, a brutality, rudeneſs, or arrogance of demeanor, ſeems, as it were, to induce a *spiteful* obſtinacy in the diſorder.

Moſt of the caſes which have proved fatal, have occurred amongſt perſons at an advanced period of life.

The old age of *nature*, and the *artificial* old age of intemperance, equally defy all remedies, *except* the dephlogiſticated nitrous gas of Dr. Beddoes; which, however, was not to be procured at the Diſpenſary.

In the caſes of phthiſis, little elſe was attempted than to relieve the troubleſome ſymptoms. In a decided inſtance of this diſeaſe, a *cure* cannot perhaps, in the preſent ſtate of medical ſcience, be reaſonably expected.

How aſtoniſhing, that in one of the moſt hopeleſs of all diſorders, Hope ſhould be one of the moſt characteriſtic ſymptoms!

There

There is no diſeaſe which more invariably and obviouſly ſhews itſelf in the complexion and general phyſiognomy of the patient.

It is remarkable, that a connection may, in many inſtances, be obſerved even between the complexion of a perſon, and his habitual occupation of life. Shoe-makers, for inſtance, are almoſt uniformly of the melancholic temperament; a circumſtance that can ſcarcely be accounted for, unleſs upon the idea that a perſon of a ſanguine diſpoſition could not eaſily reconcile himſelf to ſo ſedentary an occupation.

The fact above alluded to, which was firſt ſtated to the author of this article by his friend, Dr. Willan, has ſince been, in numerous inſtances, confirmed by his own experience. To the remarks of that learned and accurate obſerver, experience has almoſt invariably afforded a ſimilar confirmation.

